# Manipulating host secreted protein gene expression: an indirect approach by HPV11/16 E6/E7 to suppress PBMC cytokine secretion

**DOI:** 10.1186/s12985-024-02432-9

**Published:** 2024-08-02

**Authors:** Mei-zhen Zhong, Mei-nian Xu, Si-qi Zheng, Shu-qiong Cheng, Kang Zeng, Xiao-wen Huang

**Affiliations:** Department of Dermatology, Nanfang Hospital, Southern Medical University, Guangzhou, China

**Keywords:** Human papillomavirus, Peripheral blood mononuclear cells, Non-contact manner, Cytokine secretion, Secreted genes

## Abstract

Human papillomavirus (HPV) 11/16 E6/E7 proteins have been recognized to be pivotal in viral pathogenesis. This study sought to uncover the potential mechanisms of how HPV11/16 E6/E7-transfected keratinocytes inhibit cytokine secretion in peripheral blood mononuclear cells (PBMC). Upon co-culturing HPV11/16 E6/E7-transfected keratinocytes with PBMC in a non-contact manner, we observed a marked decrease in various cytokines secreted by PBMC. To determine if this suppression was mediated by specific common secreted factors, we conducted transcriptomic sequencing on these transfected cells. This analysis identified 53 common differentially secreted genes in all four HPV-transfected cells. Bioinformatics analysis demonstrated these genes were predominantly involved in immune regulation. Results from quantitative PCR (qPCR) and an extensive literature review suggested the downregulation of 12 genes (*ACE2*, *BMP3*, *BPIFB1*, *CLU*, *CST6*, *CTF1*, *HMGB2*, *MMP12*, *PDGFA*, *RNASE7*, *SULF2*, *TGM2*), and upregulation of 7 genes (*CCL17*, *CCL22*, *FBLN1*, *PLAU*, *S100A7*, *S100A8*, *S100A9*), may be crucial in modulating tumor immunity and combating pathogenic infections, with genes *S100A8* and *S100A9*, and IL-17 signaling pathway being particularly noteworthy. Thus, HPV11/16 E6/E7 proteins may inhibit cytokine secretion of immune cells by altering the expression of host-secreted genes. Further exploration of these genes may yield new insights into the complex dynamics of HPV infection.

**Supplementary Information:**

The online version contains supplementary material available at 10.1186/s12985-024-02432-9.

## Introduction

Human papillomavirus (HPV) infection, the most prevalent sexually transmitted disease, poses a significant global health risk. While HPV infection often remains asymptomatic, it can manifest in the form of cutaneous and mucosal lesions. Low-risk HPV types, specifically HPV6 and HPV11, are linked to genital warts, oral papillomas, and recurrent respiratory papillomatosis. Persistent infections with high-risk HPV strains, such as HPV16 and HPV18, can result in HPV-related carcinogenesis, including cervical, anogenital, and oropharyngeal cancers [1].

HPV infection can modify the expression of host cell genes, causing inefficient activation of the immune system. HPV E6/E7 proteins are crucial in this process. For example, E6 binding to IFN regulatory factor 3 (IRF3) [2], and E7 binding to IRF9 or IRF1 [3, 4], can suppress the expression of IFNs and interferon-stimulated genes (ISGs). These affect a range of immune cells, influencing antiviral activity, antitumor immunity, and immunomodulation [5]. HPV E7 also reduces the expression of CXCL14, which induces chemotaxis of various immune cells, including dendritic cells (DCs), natural killer (NK) cells, and T cells, thereby inhibiting the secretion of proinflammatory cytokines [6].

However, after penetrating the host’s basal layer cells via a broken surface layer of skin, HPVs remain confined to the epithelial layer throughout the infectious cycle. HPV-infected basal keratinocytes ascend through the epithelium, and ultimately, viral particles are released in the uppermost epithelial layer [7, 8]. While HPV-infected cells can regulate most immune cells’ activities, direct contact with immune cells in the epidermal layer is limited. We hypothesize that HPV-infected cells may indirectly modulate immune cell function, potentially through the expression of secreted proteins.

Recent research highlights the critical roles of secreted proteins in immune cell regulation. For instance, a group of specific secreted protein factors known as the senescence-associated secretory phenotype (SASP) can regulate macrophage polarization, recruit neutrophils, and are associated with senescence [9]. The follicular dendritic cell-secreted protein (FDCSP), mainly expressed in mucosal tissues, regulates B cell function and antibody responses [10]. Furthermore, HPV-infected keratinocytes can activate immune cells like Langerhans cells (LCs) and macrophages, and recruit effector T cells by secreting proinflammatory cytokines [8]. However, the role of other secreted proteins by HPV-infected keratinocytes remains understudied.

This study aims to identify proteins secreted by HPV11/16 E6/E7-transfected keratinocytes and to understand their impact on immune cells. We first established HPV-transfected cells and co-cultured them with peripheral blood mononuclear cells (PBMC) in a non-contact manner, confirming that the HPV-transfected cells could inhibit the secretion of various cytokines from PBMC. Consequently, we hypothesized that HPV-transfected cells might influence PBMC function in a non-contact manner, potentially through secreted proteins. Next, we performed transcriptomic sequencing on the HPV-transfected cells, screening for secreted genes that were differentially expressed in all four HPV-transfected cell types. We investigated their functions through bioinformatics analysis and validated their expression levels using quantitative PCR (qPCR) and enzyme-linked immunosorbent assay (ELISA). Finally, we reviewed the literature to analyze the potential roles of these significant genes in the non-contact regulation of PBMC function during HPV infection.

## Materials and methods

### Cell culture and cell line

Human epidermal keratinocyte line HaCaT cells and human embryonic kidney 293T cells were cultivated in Dulbecco’s Modified Eagle Medium (DMEM; Invitrogen, Carlsbad, CA, USA) with penicillin (100 U/ml), streptomycin (100 µg/ml), and 10% Fetal Bovine Serum (FBS; Invitrogen, Carlsbad, CA, USA) in a humidified incubator at 37°C with 5% CO2. Human peripheral blood mononuclear cells (hPBMC) were isolated from blood samples using the Isopaque-Ficoll method (TBD, Tianjin, China) according to the manufacturer’s instructions. Blood samples were collected from healthy volunteers, and informed consent was obtained from all subjects involved in the study. PBMC were cultured at 1 × 10^6^ cells/mL density in RPMI-1640 medium (Invitrogen, Carlsbad, CA, USA) supplemented with 10% FBS. Cells were stimulated with 5 µg/ml anti-human CD3/CD8 monoclonal antibodies (Stemcell Technologies, Vancouver, Canada) and 25 ng/mL IL-2 (PeproTech, New Jersey, USA) for 5 days and then applied for subsequent experiments.

### Generation of HaCaT cells stably expressing HPV11/16 E6/E7

As previously described [11], the *E6* and *E7* gene sequences of HPV11 (low-risk) and 16 (high-risk) were retrieved from the NCBI Nucleotide database (https://www.ncbi.nlm.nih.gov/nucleotide) and separately cloned in the pEZ-Lv203 lentiviral vector by GeneCopoeia, lnc. (Rockville, MD, USA). The plasmids of pEZ-Lv203 (empty vector), pEZ-Lv203-11-E6, pEZ-Lv203-11-E7, pEZ-Lv203-16-E6, and pEZ-Lv203-16-E7 were separately transfected into 293T cells using the Gene-Copoeia’s Lenti-Pac HIV Expression Packaging Kit following the manufacturer’s instructions. The lentiviral particles were harvested from the supernatants at 48 h post-transfection and individually infected HaCaT cells. Cells stably expressing HPV11/16 E6/E7 oncogenes were selected with 0.5 µg/ml of Puromycin antibiotic. After cultured in 6-well plates at a density of 5 × 10^5^ cells/well for 48 h, the expression of HPV11/16 E6/E7 was determined by microscopic observation of eGFP expression and qPCR, and the expression of HPV16 E6/E7 were also determined by western blot assay. HPV11/16 E6/E7-transtected cells were cultured in DMEM containing 10% FBS, penicillin (100 U/ml), streptomycin (100 µg/ml), and puromycin (2 µg/ml, Invitrogen, Carlsbad, CA, USA).

### Determination of proliferation of HPV11/16 E6/E7-expressing cells

Cell proliferation ability was analyzed by colony-forming cell assay. In brief, HPV11/16 E6/E7-expressing cells were individually seeded in 6-well plates (1000 cells per well). The empty vector-transfected cells were used as a control. After culturing for 7 days, the colonies were fixed with 4% paraformaldehyde, stained with Crystal violet dye, photographed, and counted. The proportion of cells at different cell-cycle stages was determined by propidium iodide (Biolegend, San Diego, CA, USA) staining and flow cytometry as previously reported [12].

### Co-culture of PBMC and E6/E7-transfected cells in a non-contact manner

Twelve-well transwell plates with 0.4 μm Pore Polyester Membrane Insert (Corning lnc., Corning, USA) were used for co-culture experiments. To determine the cytokine response by PBMC following a non-contact co-culture of E6/E7-transfected cells, PBMC (5 × 10^5^ cells) and E6/E7-transfected cells (5 × 10^5^ cells) were seeded into the upper and lower chambers, respectively. Co-culture of PBMC and the empty vector-transfected cells was performed as the control. The co-cultured systems were cultivated at 37°C under 5% CO2 for 24 h. Then, supernatant in the upper compartments was harvested and stored at -80℃ for subsequent detection of cytokine expression.

### Measurement of cytokine expression by PBMC

The concentration of 12 cytokines related to T-cell immunity in the supernatants of PBMC was simultaneously quantified by LEGENDplex Human Th cytokine panel (BioLegend, San Diego, CA, USA) on the flow cytometer (BD LSRFortessa, Franklin Lakes, USA) under close compliance with the manufacturer’s guidelines. Specifically, those cytokines are collectively secreted by Th1 (IL-2, IFN-γ, TNF-α), Th2 (IL-4, IL-5, IL-6, IL-9, IL-10, and IL-13), and Th17 (IL-17 A, IL-17 F, and IL-22).

### Library construction and RNA sequencing

Three replicate samples of control and HPV11/16 E6/E7-transfected cells were used for library construction and RNA sequencing, respectively. HPV11/16 E6/E7-transfected cells were disrupted directly in the TRIzol reagent (Invitrogen, Carlsbad, CA, USA). Then, the total intracellular RNA was extracted and purified according to the manufacturer’s instructions. Total RNA (1 µg per sample) was used to construct the library according to the standard procedure of Illumina. The cDNAs were subjected to the Illumina NovaSeq system in PE150 mode for sequencing. Then the sequencing data was filtered first to obtain high-quality sequencing data (Clean Data), which was compared to the reference genome of humans. Finally, the gene expression quantitative analysis, GSEA (Gene Set Enrichment Analysis), gene difference analysis, enrichment analysis, and other analyses were carried out. A threshold of FDR (False Discovery Rate) ≤ 0.05 was used to consider significant differences in gene expression.

### Bioinformatics analysis

The GO (Gene Ontology) functional annotation of GO categories, which was used to characterize the function of differentially expressed genes, and the KEGG (Kyoto Encyclopedia of Genes and Genomes) analysis method, which was performed to discover important signaling pathways of dominantly enriched genes, were performed in the Database for Annotation, Visualization, and Integrated Discovery (DAVID). The bioinformatics cloud platform (http://www.bioinformatics.com.cn/) was selected to annotate and visualize the functional involvement and pathways of differentially expressed genes. Protein-Protein Interaction Networks (PPI) were predicted by STRING database (https://string-db.org/) with confidence score more than 0.700.

### Measurement of gene expression by qPCR

HPV11/16 E6/E7-transfected cells were disrupted directly in TRIzol reagent (Invitrogen, Carlsbad, CA, USA). Then, the total intracellular RNA was extracted and purified according to the manufacturer’s instructions. The purified RNA was obtained by dissolving in RNase-free water and stored at -80°C for future use. PrimerScriptTM RT reagent Kit (TaKaRa, Otsu, Japan) was utilized to completely remove contaminating genomic DNA. RNA (1 µg) was converted to cDNA using PrimerScriptTM RT reagent Kit (TaKaRa, Otsu, Japan). Real-time PCR was quantified using Power Green qPCR Mix (GDSBio, Guangzhou, China). Primers designed for qPCR are listed in Table S1. Gene expression was examined using a Light Cycler Roche 480 PCR instrument with the following protocol: initial denaturation at 95°C for 1 min, followed by 40 cycles of 95°C for 5 s and 60°C for 34 s, then ending by a melt-curve analysis of 60°C to 95°C with the instrument default setting. The mRNA levels of target genes were obtained using the 2^−△△Ct^ method and normalized to *GAPDH* expression. Assays were performed with three independent experimental samples in triplicates.

### Cell supernatant secreted protein measurements

The levels of seven human secreted proteins S100A7, S100A8, S100A9, CCL17, CCL22, BPIFB1, and CLU were determined in cell supernatant samples from transfected cells using corresponding ELISA kits (Ruixin Biotech, Quanzhou, China; Website: ruixinbio.com) according to the manufacturer’s instructions.

### Western blot assay

In preparation, cells were washed twice with cold PBS and lysed using the RIPA lysis buffer containing phosphatase and protease inhibitors (FDbio Science Biotech Co., Ltd., Hangzhou, China) on ice for 10 min and then centrifuged at 12,000 rpm at 4 °C for 10 min to obtain the supernatant for western blot analysis. Primary antibodies against HPV16 E6 (Abcam, USA), HPV16 E7 (Abcam, USA), phospho-p38/p38 (Cell Signaling Technology, USA) and ACTB (Cell Signaling Technology, USA) were used.

### Statistical analysis

We used GraphPad Prism version 9 (GraphPad Software, San Diego, CA) for the data analysis and statistical tests of cytokine secretion by PBMC, and gene expression of keratinocytes by qPCR and ELISA. Mean values and standard deviations were compared using a one-way ANOVA. The data were considered statistically significant at a *P* value of <0.05.

## Results

### HPV11/16 E6/E7 transfection promoted the proliferation of HaCaT cells

**Fig. 1:**
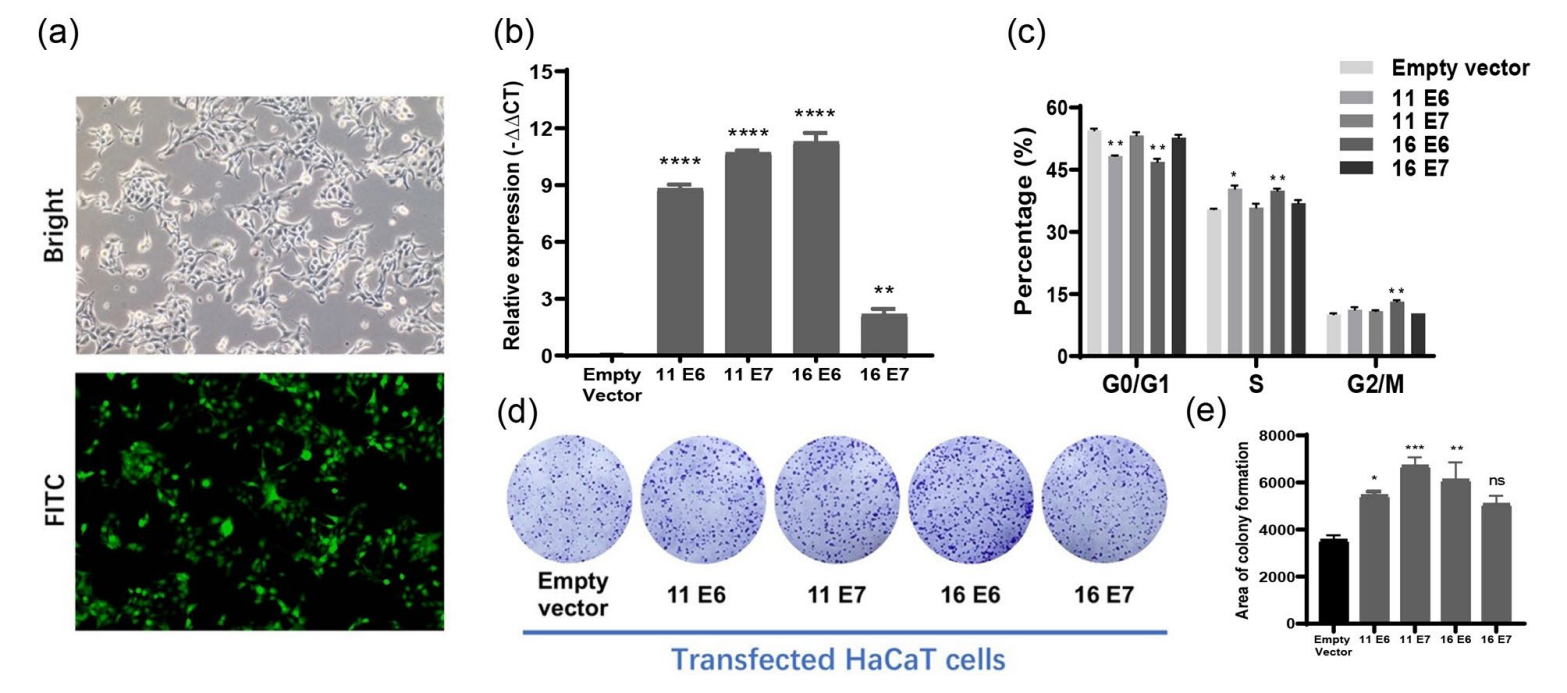
Construction of HaCaT cells stably expressing HPV11/16 E6/E7. **a** Observation of green fluorescence in the transfected cells containing vectors fused with eGFP reporter gene. **b** Relative expression of HPV11/16 E6/E7 mRNAs in transfected cells. **c** Analysis of cell-cycle stages of transfected cells by flow cytometry. **d** Colony formation capacity of transfected cells after culturing for 7 days. **e** The quantification of colony formation capacity of transfected cells after culturing for 7 days. *, *p* < 0.05; **, *p* < 0.01; ****, *p* < 0.0001

First, we established HaCaT cells stably expressing HPV11/16 E6/E7. As shown in Fig. 1a and Figure S1a, green fluorescence was observed in transfected HaCaT cells containing the vectors fused with the eGFP reporter gene. The mRNA expression of HPV11 E6, HPV11 E7, HPV16 E6, and HPV16 E7 significantly increased in the transduced HaCaT cells compared to the control (empty vector-transfected cells), indicating overexpression of HPV11/16 E6/E7 genes (Fig. 1b, *p* < 0.05). Flow cytometry analysis revealed that HPV11 E6 and HPV16 E6 overexpression resulted in a reduced cell proportion in the G0/G1 phase, with a concomitant increase in the percentage of cells in the S-phase. Meanwhile, HPV16 E6-expressing cells accumulated in the G2/M phase. No significant cell cycle variations were observed in both HPV11 E7 and HPV16 E7-expressing cells compared to those transfected with the empty vector (Fig. 1c). These results suggest that HPV11 E6 and HPV16 E6 induced a proliferating state and DNA synthesis. Next, we employed the colony formation assay to detect cell proliferation. As evident in Fig. 1d and e, HPV11/16 E6/E7 expression enhanced the colony formation capacity of HaCaT cells, indicating that HPV11/16 E6/E7 promoted cell proliferation.

### HPV11/16 E6/E7-transfected cells suppressed comprehensive secretion of Th-related cytokines

**Fig. 2:**
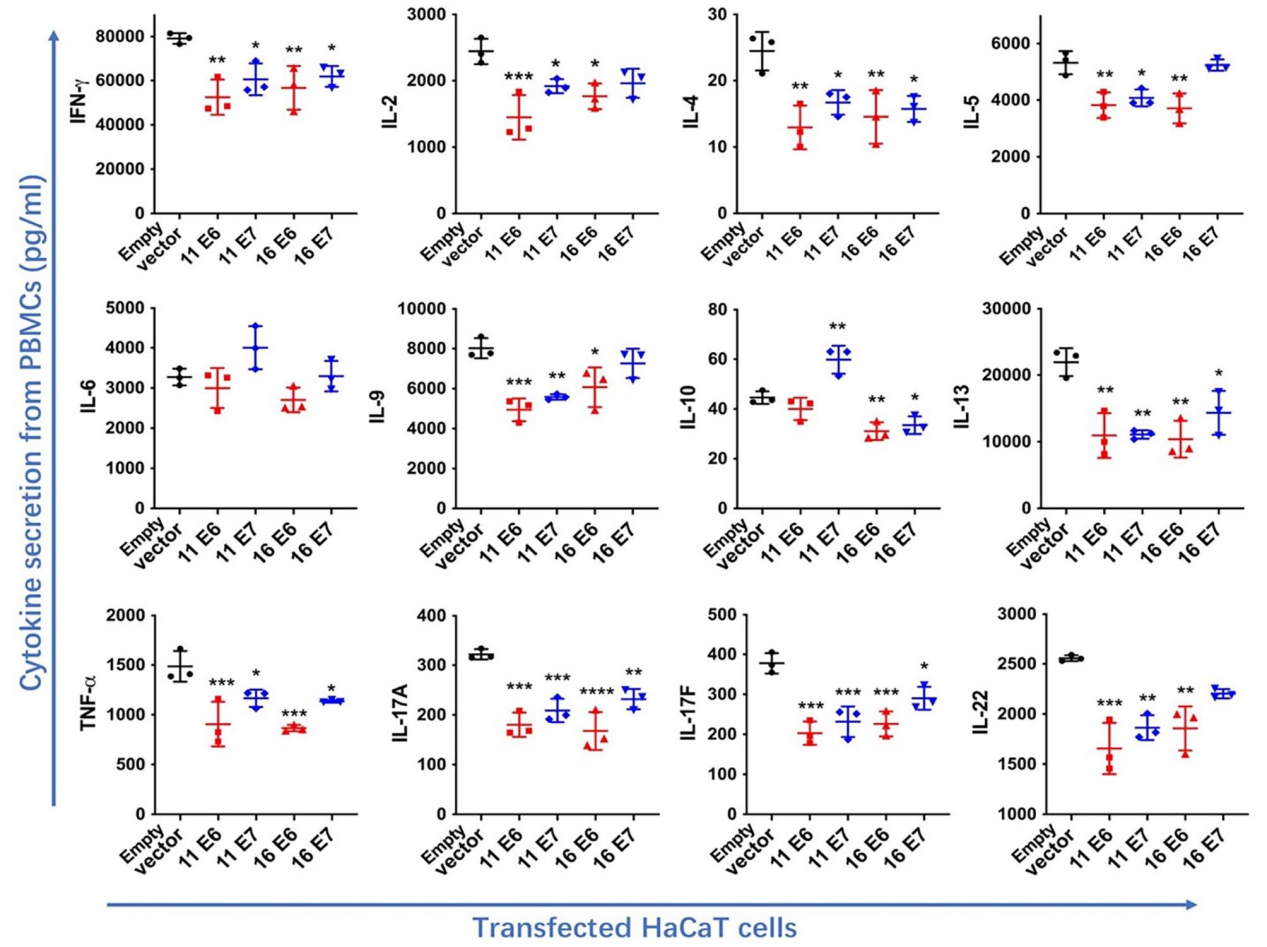
Cytokine expression in the supernatants of PBMC after non-contacting co-culture with HPV11/16 E6/E7-transfected cells for 24 h. Co-culture of PBMC and the empty vector-transfected cells was performed as the control. Values are means ± standard errors of the means (SEM). *, *p* < 0.05; **, *p* < 0.01; ***, *p* < 0.001; ****, *p* < 0.0001. One-way ANOVA test with Bonferroni’s correction

Previous research has shown that HPV facilitates an immuno-suppressive environment by expressing E6 and E7 proteins. Cytokines are essential mediators of immune responses. PBMC primarily consist of lymphocytes (T cells, B cells, and NK cells), phagocytes, and DCs. We harvested the supernatants from PBMC to detect Th-related cytokines after non-contacting co-culture with HPV11/16 E6/E7-transfected cells. As illustrated in Fig. 2, HPV 11/16 E6/E7-transfected cells significantly suppressed the secretion of IFN-γ, IL-4, IL-13, TNF-α, IL-17 A, and IL-17 F (*p* < 0.05) compared to the empty vector-transfected group. PBMC co-culture with HaCaT cells transfected with HPV11 E6, HPV11 E7, or HPV16 E6 significantly decreased production of IL-2, IL-5, IL-9, and IL-22 (*p* < 0.05). Furthermore, IL-10 secretion from PBMC was significantly suppressed by co-culture with HPV 16 E6/E7-transfected cells.

### HPV11/16 E6/E7-transfected cells expressed immunomodulatory genes

**Fig. 3:**
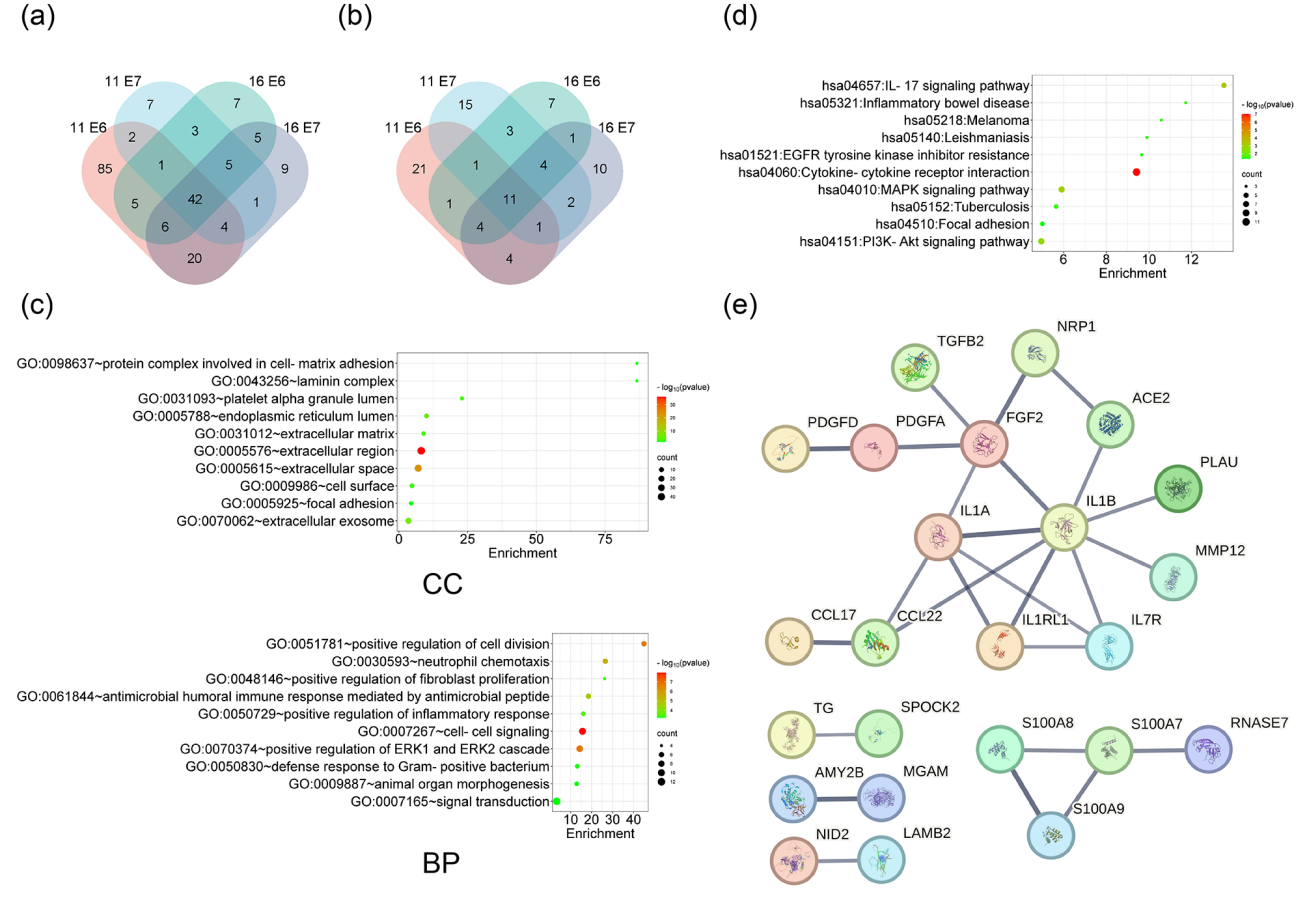
Expression profiles of mRNAs in HPV11/16 E6/E7-expressing cells compared to the empty vector-transfected cells. **a** Venn diagram of the downregulated secreted genes in HPV11/16 E6/E7-expressing cells. **b** Venn diagram of the upregulated secreted genes in HPV11/16 E6/E7-expressing cells. **c** GO enrichment analysis of cellular component (CC, TOP 10) and biological process (BP, TOP 10) of the commonly differentially expressed secretory genes in HPV11/16 E6/E7-expressing cells. **d** KEGG pathway enrichment analysis of commonly differentially expressed secretory genes (TOP 10) in HPV11/16 E6/E7-expressing cells. **e** Protein-Protein Interaction Networks (PPI) of the commonly differentially expressed secretory genes in HPV11/16 E6/E7-expressing cells at high-confidence (score 0.700)

To further investigate the non-contact inhibition mechanism of PBMC by HPV11/16 E6/E7-transfected cells, we aimed to measure the mRNA expression profiles in HPV11/16 E6/E7-expressing cells and identify differentially expressed secreted genes in all four HPV-transfected cell types compared to the empty vector-transfected cells. As depicted in Fig. 3a and b, we detected 42 commonly downregulated secreted genes and 11 commonly upregulated secreted genes across all four types of HPV-transfected cells. These genes are listed in Table 1. GO enrichment analysis showed that the proteins encoded by these genes are primarily extracellular fragments or directly secreted outside the cells (Fig. 3c, CC, cellular component), playing crucial roles in regulating immune responses (Fig. 3c, BP, biological process). KEGG pathway enrichment analysis revealed that these genes were mainly involved in immunity-regulating pathways, including cytokine-cytokine receptor interaction, IL-17 signaling pathway, MAPK signaling pathway, and PI3K-Akt signaling pathway (Fig. 3d). To further examine the interactions of the proteins encoded by these genes, Fig. 3e demonstrated that most of them may interact with other proteins.

**Table 1:** The commonly differentially expressed secretory genes in HPV11/16 E6/E7-transfected cells

Expression	Genes
Downregulation	*ACE2*, *AMY2B*, *ARTN*, *BMP3*, *BPIFB1*, *CCL17*, *CCL22*, *CD74*, *CLEC3A*, *CLU*, *CST6*, *CTF1*, *ECM2*, *FBLN1*, *FGFBP3*, *GDF15*, *IGFBP3*, *IL1A*, *IL1B*, *ISLR*, *LAMB2*, *MGAM*, *MMP12*, *NTN4*, *PDGFA*, *PDGFD*, *PGLYRP4*, *PLAU*, *PSAPL1*, *PSG5*, *RNASE7*, *S100A7*, *S100A8*, *S100A9*, *SPOCK2*, *STC2*, *SULF2*, *TG*, *TGFB2*, *TGM2*, *TNFSF15*, *TNXB*
Upregulation	*C18ORF54*, *CEMIP*, *CLCA2*, *FGF2*, *HMGB2*, *IL1RL1*, *IL7R*, *LGALS1*, *MFGE8*, *NID2*, *NRP1*

### Validation of the commonly differentially expressed secretory genes

**Fig. 4:**
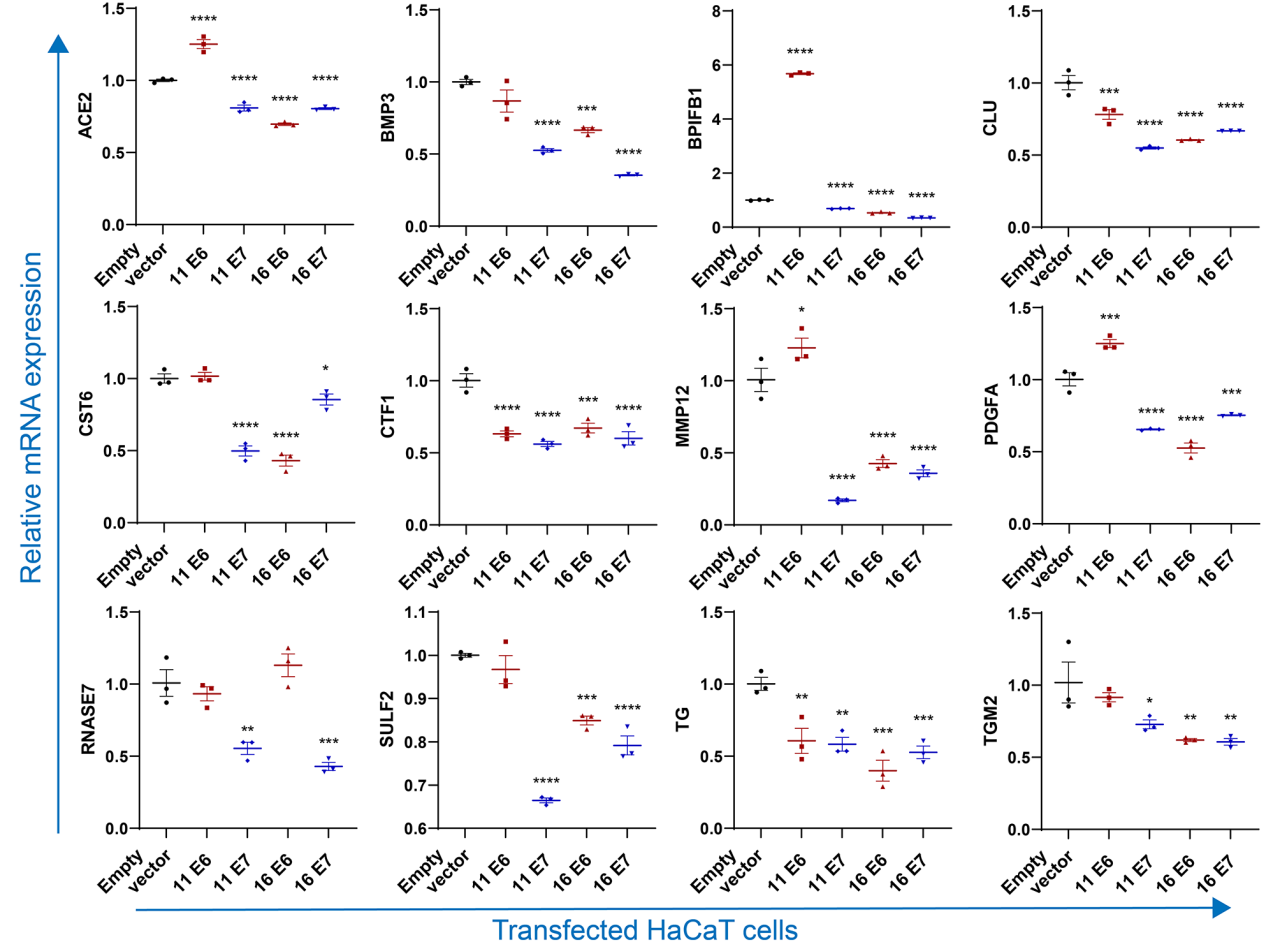
Gene expression of HPV11/16 E6/E7-transfected HaCaT cells confirmed by qPCR. An empty vector-transfected cell was used as the control. Values are means ± standard errors of the means (SEM). *, *p* < 0.05; **, *p* < 0.01; ***, *p* < 0.001; ****, *p* < 0.0001. One-way ANOVA test with Bonferroni’s correction

**Fig. 5:**
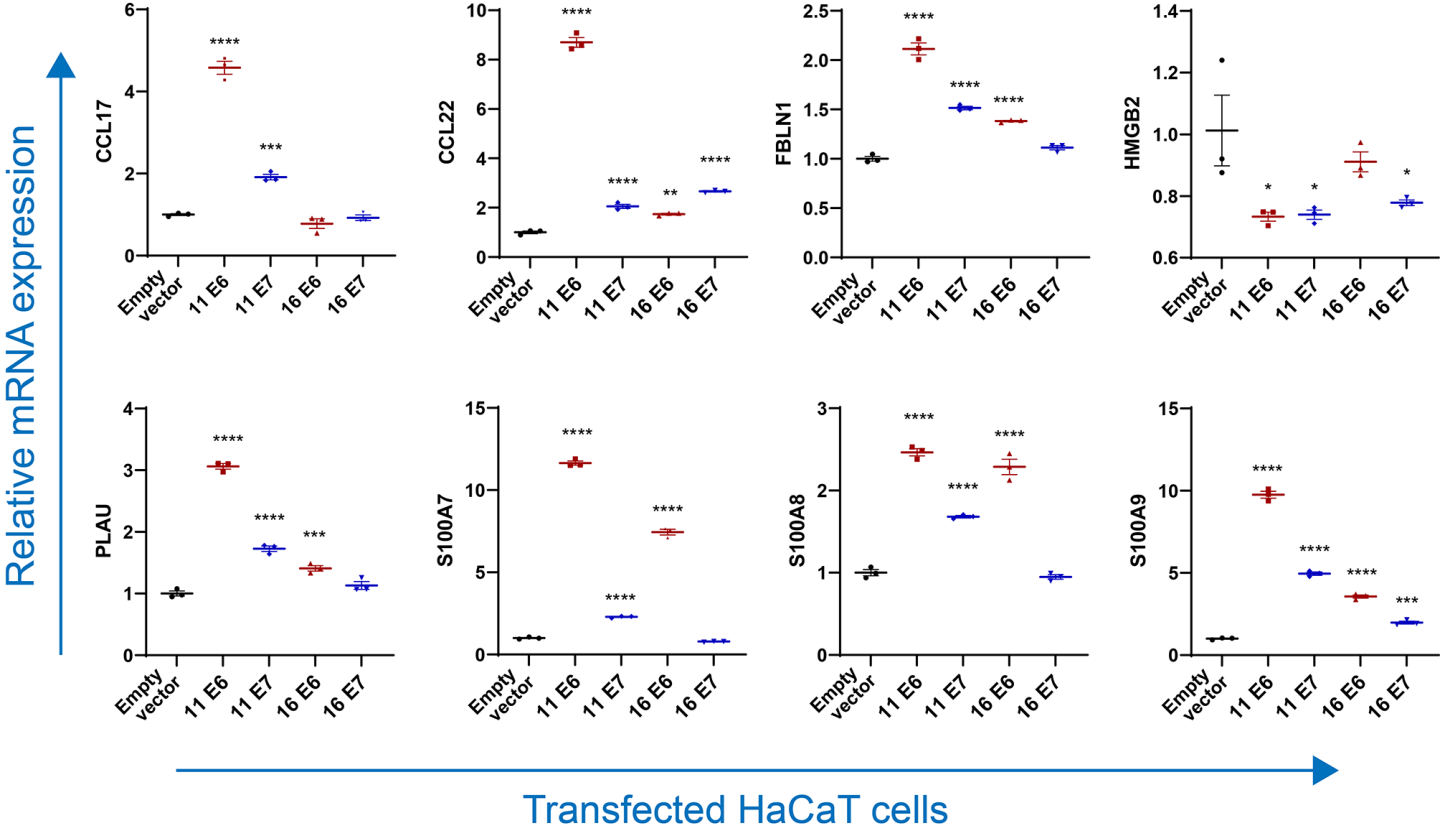
Gene expression of HPV11/16 E6/E7-transfected HaCaT cells by qPCR, the results of which were in contrast to sequencing results. An empty vector-transfected cell was used as the control. Values are means ± standard errors of the means (SEM). *, *p* < 0.05; **, *p* < 0.01; ***, *p* < 0.001; ****, *p* < 0.0001. One-way ANOVA test with Bonferroni’s correction

**Fig. 6:**
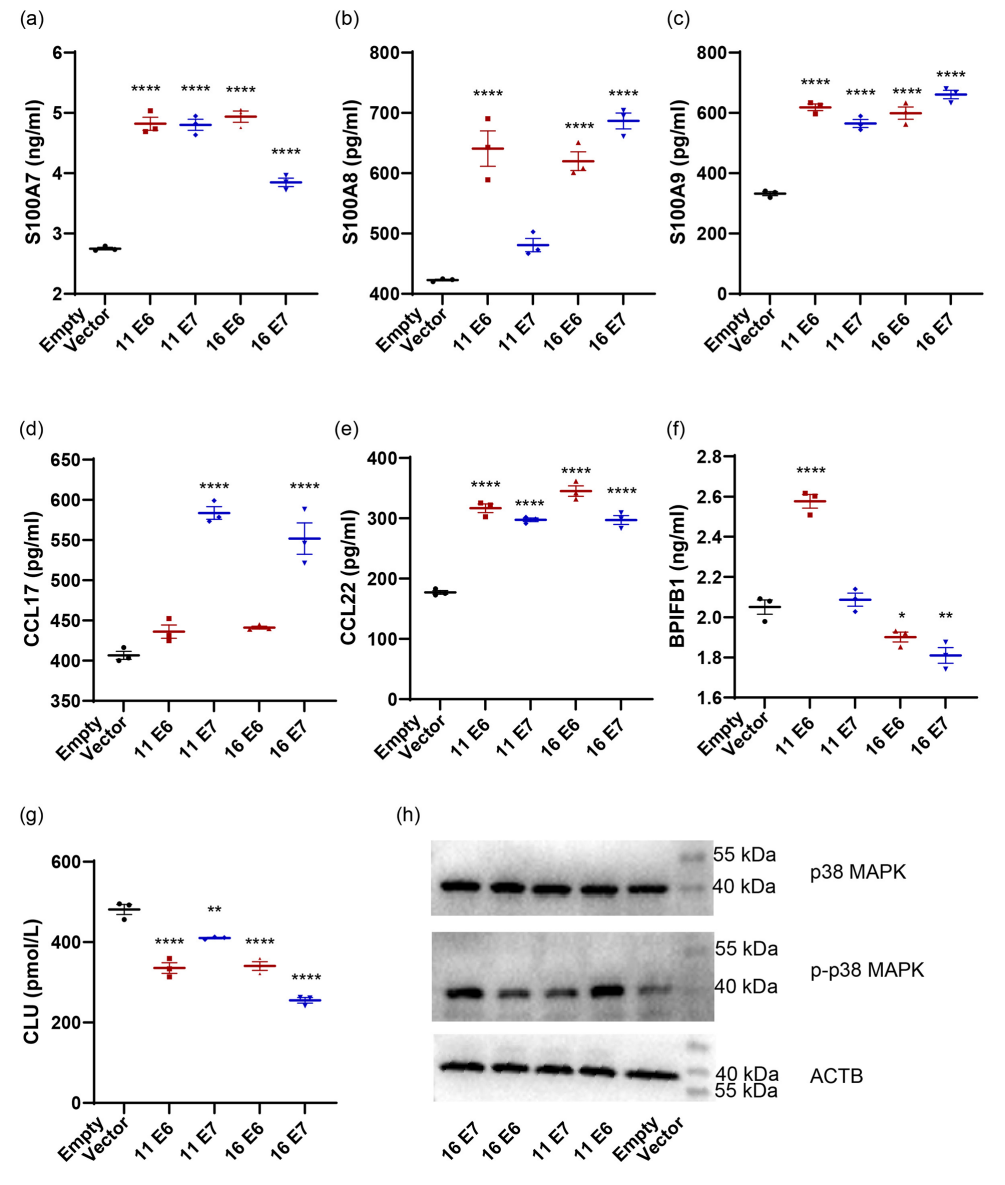
**a-g** Secreted protein production of cell supernatant from HPV11/16 E6/E7-transfected HaCaT cells by ELISA. **h** The protein levels of phospho-p38/p38 MAPK and ACTB in HPV11/16 E6/E7-transfected HaCaT cells by WB. An empty vector-transfected cell was used as the control. Values are means ± standard errors of the means (SEM). *, *p* < 0.05; **, *p* < 0.01; ***, *p* < 0.001; ****, *p* < 0.0001. One-way ANOVA test with Bonferroni’s correction

To further validate the expression profiles of mRNAs in HPV11/16 E6/E7-transfected cells, cells were harvested to detect the common differentially secreted genes by qPCR. As shown in Fig. 4, compared with the empty vector-transfected group, HPV 11/16 E6/E7-transfected cells significantly suppressed the secretion of *CLU*, *CTF1*, and *TG* (*p* < 0.05). HaCaT cells transfected with HPV11 E7, HPV16 E6, and HPV16 E7 significantly decreased expression of *ACE2*, *BPIFB1*, *BMP3*, *CST6*, *MMP12*, *PDGFA*, *SULF2*, and *TGM2* (*p* < 0.05), which were found no significant differences or upregulation in HPV11 E6-transfected cells. However, in contrast to the sequencing results, HaCaT cells transfected with HPV11 E6, HPV11 E7, and HPV16 E6 significantly upregulated the expression of *CCL22*, *FBLN1*, *PLAU*, *S100A7*, *S100A8*, and *S100A9* (*p* < 0.05), which were found no significant differences or upregulation in HPV16 E7-transfected cells (Fig. 5). To further validate the production of some important secreted proteins, cell supernatant samples from these transfected cells were detected. As shown in Fig. 6, HaCaT cells transfected with HPV11 E6, HPV11 E7, and HPV16 E6 significantly upregulated the production of CCL22, S100A7, and S100A9, and significantly downregulated the production of CLU. Importantly, the protein phospho-p38 MAPK involves in IL-17 signaling pathway also upregulated in all four HPV-transfected cells.

Based on the qPCR results, we further explored the potential roles of these genes in HPV infection by reviewing the literature. As shown in Table 2, genes such as *ACE2*, *BMP3*, *BPIFB1*, *CCL17*, *CCL22*, *CLU*, *CST6*, *CTF1*, *FBLN1*, *HMGB2*, *MMP12*, *PDGFA*, *PLAU*, *RNASE7*, *S100A7*, *S100A8*, *S100A9*, *SULF2*, and *TGM2* primarily function in regulating immune responses to exert anti-tumor and anti-infection effects. This suggests that HPV11/16 E6/E7 may suppress PBMC cytokine secretion by modulating the expression of these host cell-secreted genes. KEGG pathway enrichment analysis revealed that these genes were mainly involved in pathways such as the IL-17 signaling pathway, and cytokine-cytokine receptor interaction, with *BMP3*, *CCL17*, *CCL22*, *CTF1*, *S100A7*, *S100A8*, and *S100A9* being central components.

**Table 2:** Potentially important genes in HPV infection

Gene	Potential roles in HPV infection
*ACE2*	Inhibit tumor angiogenesis [13] Promote tumor immune infiltration [14] Induce proinflammatory cytokine expression [15]
*BMP3*	Regulate tumor immunity [16]
*BPIFB1*	Delay cell cycle progression from G1 to S phase [17] Activate the macrophage M2 polarization [18]
*CCL17*	Regulate recruitment of regulatory T cells (Treg) [19–21] Promote the recruitment of IL-17 secreting T cells [22] Attenuate CCL5-induced mast cell migration [23]
*CCL22*	Induce proinflammatory changes [24] Facilitate the infiltration of Treg [21] Promote the recruitment of IL-17 secreting T cells [22] Attenuate CCL5-induced mast cell migration [23]
*CLU*	Activate immune-related pathways against pathogenic infections [25] Promote immune cell infiltration [26] Promote the expansion and IFN-γ production of natural killer cells [27]
*CST6*	Regulate tumor immunity [28]
*CTF1*	Regulate tumor immunity [29] Promote autophagy, affecting immune cells [30]
*FBLN1*	Increase expression of inflammatory cells and proinflammatory cytokines/chemokines [31]
*HMGB2*	Regulate differentiation and function of memory CD8^+^ T cells [32] Promote expression of type-I IFNs and inflammatory cytokines [33]
*MMP12*	Increase Treg infiltration [34] Polarize neutrophils towards a strong apoptotic signature [35] Inhibit M2 macrophage accumulation [36]
*PDGFA*	Regulate the immune microenvironment [37, 38]
*PLAU*	Modulate the functions of immune cells [39]
*RNASE7*	Regulate innate immune against pathogenic infections [40] Downregulate TH2 cytokine production by activated human T cells [41]
*S100A7*	Enhance immunosuppressive tumor microenvironment [42]
*S100A8*	Recruit Myeloid-derived suppressor cells (MDSCs) [43]
*S100A9*	Recruit MDSCs [43] Activate inflammation and enhance HPV oncogene expression [44]
*SULF2*	Regulate the antigen presentation and phagocytic activities of macrophages [45] Mediate the TNF-α-induced inflammatory activation [46]
*TGM2*	Activate CD8^+^ T cells and increase expression of IFN-γ to activate antitumor immunity [47]

## Discussion

In this study, we observed significant suppression of various cytokine secretion by PBMC when co-cultured with HPV11/16 E6/E7-transfected cells in a non-contact manner. To further investigate the potential mechanisms underlying the suppression of cytokine secretion in PBMC by HPV11/16 E6/E7-transfected keratinocytes, we identified commonly differentially expressed secretory genes using transcriptomic sequencing. GO and KEGG pathway enrichment analysis revealed that these genes are primarily involved in immune regulatory biological processes and immune signaling pathways. The PPI networks suggests that the secreted proteins encoded by these genes also have complex interactions. Results from qPCR, ELISA and an extensive literature review suggested that secreted genes, including *ACE2*, *BMP3*, *BPIFB1*, *CCL17*, *CCL22*, *CLU*, *CST6*, *CTF1*, *FBLN1*, *HMGB2*, *MMP12*, *PDGFA*, *PLAU*, *RNASE7*, *S100A7*, *S100A8*, *S100A9*, *SULF2*, and *TGM2*, may play critical roles in mediating immune cell functions and cytokine expression.

HPV11/16 E6/E7-transfected cells inhibited the secretion of various cytokines by PBMC, including IFN-γ, IL-2, IL-4, IL-5, IL-9, IL-10, IL-13, TNF-α, IL-17 A, IL-17 F, IL-22. These cytokines, secreted by various immune cells from both innate and adaptive immune systems, play crucial roles in modulating viral replication and host immune responses following HPV infection. IFN-γ enhances the antiviral immune response, exerting direct antiviral functions [48]. IL-2 promotes the cytolytic activity of CD8^+^ T cell and NK cells and modulates the expansion and differentiation of Th-1, Th-2, Th-17, and Tregs lineages [49], potentially exerting antiviral functions. Th2 cytokines, including IL-4, IL-5, IL-9, IL-10, and IL-13, primarily function by inhibiting acute inflammation [50]. Moreover, IL-4 can downregulate the oncogene expression of HPV16 [51]. TNF-α, a proinflammatory cytokine, is essential for inducing apoptosis in HPV-infected cells [52]. Th17 cytokines (IL-17 A, IL-17 F, IL-22) also exert proinflammatory effects, and can suppress the proliferation of HPV-associated epithelial cells [53]. In conclusion, these cytokines may exert anti-inflammatory or proinflammatory effects, ultimately inhibiting viral replication or suppressing the survival of infected cells. The reduction of these cytokines, indicative of immune escape by HPV, may be beneficial for the exacerbation of infection or tumor progression.

HPV-infected cells modulate PBMC cytokine secretion in a non-contact manner primarily through paracrine control, including the secretion of growth factors, cytokines, chemokine, or extracellular matrix [54]. Recent studies have highlighted the importance of cytokines in HPV-infected cells. For instance, high-risk HPV-infected cells enhance IL-6 expression, which modulates the tumor immune microenvironment via paracrine signaling transduction [55]. However, other paracrine components from HPV-infected keratinocytes remain underexplored. This study identified several novel factors, including the downregulation of 12 genes (*ACE2*, *BMP3*, *BPIFB1*, *CLU*, *CST6*, *CTF1*, *HMGB2*, *MMP12*, *PDGFA*, *RNASE7*, *SULF2*, *TGM2*), and upregulation of 7 genes (*CCL17*, *CCL22*, *FBLN1*, *PLAU*, *S100A7*, *S100A8*, *S100A9*). These genes, encompassing cytokines, enzymes, glycoproteins, growth factors, and antimicrobial peptides, play critical roles in tumor immunity regulation and pathogenic infections resistance by modulating immune cells functions and cytokine secretion.

*BPIFB1*, involved in innate immune response in mucosa and negative regulation of toll-like receptor 4 signaling pathway [56], activates M2 macrophage polarization [18], which exerts anti-inflammatory effects by activating Th2 cells and releasing anti-inflammatory factors (IL-1, IL-10, and TGF-β). *CTF1* promotes autophagy [30], which may critically regulate various immune cells functions via pathways including cytokine-cytokine receptor interaction and JAK-STAT signaling pathway. The reduction of *BPIFB1* and *CTF1* may therefore contribute to the downregulation of various cytokines secreted by immune cells. Notably, *S100A8* and *S100A9*, involved in the IL-17 signaling pathway, can recruit myeloid-derived suppressor cells (MDSCs) [43], crucial for inhibiting T cell activation [57]. Furthermore, a study showed that *S100A8* and *S100A9* can regulate psoriasis by inhibiting production of the IL-17 A and IL-17 F [58]. Thus, we hypothesize that the increase of *S100A8* and *S100A9*, the myeloid-related damage-associated molecular patterns (DAMPs), after HPV infection, may recruit MDSCs to inhibit various cytokine secretion. Moreover, the upregulation of protein phospho-p38 MAPK involves in IL-17 signaling pathway further verified the S100A8/S100A9-MAPK cascade may be critical in regulating cell proliferation and apoptosis [59, 60].

However, the roles of several genes, such as *CCL17* and *CCL22*, appear contradictory. These genes are involved in pathways including cytokine-cytokine receptor interaction, viral protein interaction with cytokine and cytokine receptor, chemokine signaling pathway, C-type lectin receptor signaling pathway, and IL-17 signaling pathway. The upregulation of *CCL17* and *CCL22* can facilitate Treg infiltration [21] and attenuate mast cells migration [23], both of which involve suppressing various cytokine secretion. However, they can also promote the recruitment of IL-17 secreting T cells [22], while *CCL17* can also suppress Treg infiltration [19]. In conclusion, HPV11/16 E6/E7-transfected cells may suppress PBMC cytokine secretion by modulating their secreted gene expression, with genes *S100A8* and *S100A9*, and the IL-17 signaling pathway, being particularly noteworthy.

This study mainly discussed the common roles of HPV11/16 E6/E7 proteins in suppressing cytokine secretion from immune cells by inhibiting the expression of host cell secretory gene. However, as high-risk types have the potential to cause cancer, while low-risk types do not, there must be a significant difference in pathogenesis between high-risk HPV16 and low-risk HPV11, which is worthwhile to further investigate. Compared with low-risk HPV types, besides regulating inflammation, high-risk HPV strains might play more important roles in regulating cell cycle and apoptosis, which is critical for carcinogenesis. As shown by qPCR and ELISA, BPIFB1 increased in HPV11 E6-transfected cells but decreased in HPV16-transfected cells. Moreover, a study showed that secreted protein BPIFB1 can inhibit LPS stimulated nasopharyngeal epithelium cell proliferation, and prevent carcinogenesis of nasopharyngeal carcinoma [61]. We speculated that BPIFB1 may involve in the pathogenesis of cervical cancer.

## Conclusion

This study unveiled the potential mechanism by which HPV11/16 E6/E7 proteins suppress cytokine secretion from immune cells by inhibiting the expression of host cell secretory gene, offering fresh insights into HPV-induced immunosuppression. However, the specific roles of these genes warrant further investigation. Future studies will be conducted in this regard.

### Electronic supplementary material

Below is the link to the electronic supplementary material.

Table S1: Primers designed for qPCR. Figure S1: a The expression of HPV11/16 E6/E7 determined by microscopic observation of eGFP expression. b The expression of HPV16 E6/E7 determined by western blot assay

## Data Availability

No datasets were generated or analysed during the current study.

## Abbreviations

HPV: Human papillomavirus
PBMC: Peripheral blood mononuclear cells
qPCR: Quantitative PCR
IRF: IFN regulatory factor
ISGs: Interferon-stimulated genes
DCs: Dendritic cells
NK: Natural killer
SASP: Senescence-associated secretory phenotype
FDCSP: Follicular dendritic cell-secreted protein
LCs: Langerhans cells
ELISA: Enzyme-linked immunosorbent assay
GSEA: Gene Set Enrichment Analysis
FDR: False Discovery Rate
GO: Gene Ontology
KEGG: Kyoto Encyclopedia of Genes and Genomes
DAVID: Database for Annotation, Visualization, and Integrated Discovery
PPI: Protein-Protein Interaction Networks
Treg: Regulatory T cells
MDSCs: Myeloid-derived suppressor cells
DAMPs: Damage-associated molecular patterns
