# Supplementary material for: Manipulating host secreted protein gene expression: an indirect approach by HPV11/16 E6/E7 to suppress PBMC cytokine secretion
Source: Virol J. 2024 Aug 2;21:172. doi: 10.1186/s12985-024-02432-9 (PMC11295672; doi:10.1186/s12985-024-02432-9)

**Table S1.** Primers designed for qPCR.

| Gene | Forward primer | Reverse primer |
| --- | --- | --- |
| *ACE2* | ATGGCTATGACTACAGCCGC | ACCCCACATATCACCAAGCA |
| *AMY2B* | GGGAGAAGGTTGGGGTTTCAT | ACTGCCATTTTATACAGCCTAGC |
| *ARTN* | ACACCCGAGGGTGCAGA | GCCTCCAAGTCCAAGTTCCA |
| *BMP3* | GCGGCAGCAGCAGAAACTC | GGGAGCATCCTCCAGACACT |
| *BPIFB1* | CCTTCCTGGTGAACGCCTTA | AGGGAAATGGGCACCTTCAC |
| *CCL17* | CCAGGGATGCCATCGTTTTTG | TAGTCCCGGGAGACAGTCAG |
| *CCL22* | ACAGACTGCACTCCTGGTTG | CCCTGAAGGTTAGCAACACCA |
| *CD74* | AGAATGCTGACCCCCTGAAG | CACTTGGTCAGTACTTTCGGT |
| *CLEC3A* | CAAACGTCGAGTGAGAGACAAG | TGCAGTCTTCATTGGCCTCAT |
| *CLU* | GTCACTGAGGTGGTCGTGAA | ACATCTCACTCCTCCCGGT |
| *CST6* | GACACGCACATCATCAAGGC | CACAGCGCAGCTTCTCCT |
| *CTF1* | AGGGAAGTCTGGAAGACCCC | AGCTGCTCAGCGTATTTGGT |
| *ECM2* | GTGTTTGGCCACATGGAACC | CACTTGGTCAGTACTTTCGGT |
| *FBLN1* | CCGCAACTGCCAAGACATT | CTTGGAGCACTCCCGATTCT |
| *FGFBP3* | CCTCTCCCAGAACTCGGTCC | AGCTTCGGAGGAGTCATGC |
| *GDF15* | ACTCACGCCAGAAGTGCG | GTCACGTCCCACGACCTTG |
| *IGFBP3* | CCGCGCCAGGAAATGCT | AACTTGGGATCAGACACCCG |
| *IL1A* | GCGTTTGAGTCAGCAAAGAAGT | CATGGAGTGGGCCATAGCTT |
| *IL1B* | AACCTCTTCGAGGCACAAGG | GTCCTGGAAGGAGCACTTCAT |
| *ISLR* | TGACCTCGGGAGGCACC | GTAGTCACATTGGCCGGGAA |
| *LAMB2* | CAGAGTTGACACGGAACCCC | GGCAGCCAGCACGCTTA |
| *MGAM* | GCAAGAGGTAATGAGAGATGGCA | GACCCGTTGTCCTAGCATGT |
| *MMP12* | GGCCCGTATGGAGGAAACAT | GGAAGTCTCCATGAGCTCCAC |
| *NTN4* | ATGCTTGCAAACCGTGTTCC | GTCGACAGCCATAGTCTCCG |
| *PDGFA* | CAGCGACTCCTGGAGATAGAC | GGACAGCTTCCTCGATGCTT |
| *PDGFD* | GCAGAACCCGGCTTTTTCTT | GGTGATTGCTCTCATCTCGC |
| *PGLYRP4* | CTGCCGTGGCTTCTTGTCTT | CATCTGTGGGAAGGCCTTTTTC |
| *PLAU* | AGCGACTCCAAACGAACTG | TCAGTGCTGGCCTTTCCTC |
| *PSAPL1* | ACCAAGCACTGAGGCTTCTC | TCATGCACACCTCACAGGTC |
| *PSG5* | AGTCGAAGTCTCTGCTCCTT | TGTCTGCCAGTCTTCCTGA |
| *RNASE7* | CCAAGCGCAAAGCGACC | GCTGAGTTGCATGCTTGAGG |
| *S100A7* | CACTCAAGCTGAGAGGTCCA | ACATCGGCGAGGTAATTTGTG |
| *S100A8* | GTTCTGTTTTTCAGGTGGGGC | CGTCTGCACCCTTTTTCCTGA |
| *S100A9* | GGCTCCTCGGCTTTGACAG | GCACCAGCTCTTTGAATTCCC |
| *SPOCK2* | TGCCAGAAGGTGAAGTGCAG | TGCTCCAGCTTACACACAGAG |
| *STC2* | TGAAATGTAAGGCCCACGCT | GTCCACGTAGGGTTCGTGC |
| *SULF2* | GAGAGGCAAGCTGCTACACA | ACACACTGCCACTTCTGTCC |
| *TG* | CAGACTGTCCAGTGCCAGAA | GGGAGGTAGGAGGTGTCTGT |
| *TGFB2* | ATCCTGAGCCCGAGGAAGTC | ACTGGGCAGACAGTTTCGGA |
| *TGM2* | GTTTGGGGAGATCCAGGGTG | CCACAGCAGTACGTCCCTTC |
| *TNFSF15* | GAGCTTTGGGGAAACAGCCA | TCCTGTCCTTTTAGAGCCTGGA |
| *TNXB* | CACAGTGGACCATCAGGGAG | TCAGTGCTCGGCAGTCATAC |
| *C18ORF54* | CTACACTTCCATGGGTGACAA | CCCTTTAGCACTCTTTGCCCT |
| *CEMIP* | ACAGGCAGGACAACGGGAG | AAGAGGTGAGCAGCAGTGTC |
| *CLCA2* | ACTGTGGGCAACGACACTAT | GTCCAGTGCCCAGGCTTAG |
| *FGF2* | GAGCGACCCTCACATCAAGC | TGCCCAGTTCGTTTCAGTGC |
| *HMGB2* | ACTCTGAGGAAAAGCTCGCA | TTTGCAGACATGGTCTTCCATC |
| *IL1RL1* | TGACTGAGGACGCAGGTGAT | AAGCCTTGCTCATCCTTGACC |
| *IL7R* | TGGCTATGCTCAAAATGGAGACT | CAGGCACTTTACCTCCACGA |
| *LGALS1* | CCTGACGCTAAGAGCTTCGT | GGAAGGGAAAGACAGCCTCC |
| *MFGE8* | AGGGCCAGGACTCTTTAGGT | AGTGGCTCGACACATTTCGT |
| *NRP1* | CGAGGGCGAAATCGGAAAAG | GGCGTGCTCCCTGTTTCA |
| *GAPDH* | GTCTCCTCTGACTTCAACAGCG | ACCACCCTGTTGCTGTAGCCAA |

Figure S1: a The expression of HPV11/16 E6/E7 determined by microscopic observation of eGFP expression. b The expression of HPV16 E6/E7 determined by western blot assay


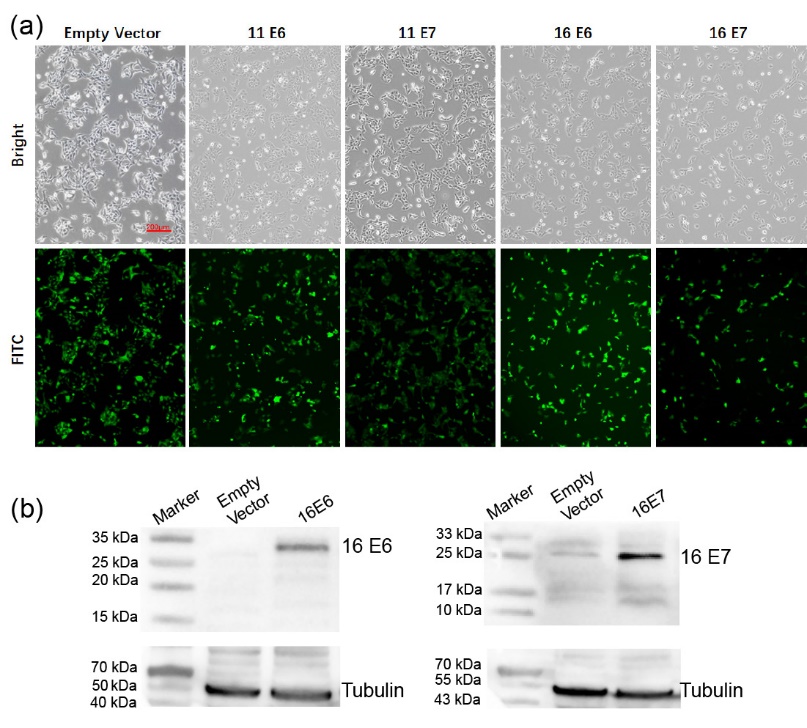

Supplement: Supplementary file 1 — Table S1: Primers designed for qPCR. Figure S1: a The expression of HPV11/16 E6/E7 determined by microscopic observation of eGFP expression. b The expression of HPV16 E6/E7 determined by western blot assay [file 12985_2024_2432_MOESM1_ESM.docx]
